# Biochemical Defense Response: Characterizing the Plasticity of Source and Sink in Spring Wheat under Terminal Heat Stress

**DOI:** 10.3389/fpls.2017.01603

**Published:** 2017-09-20

**Authors:** Ranjeet R. Kumar, Suneha Goswami, Mohammed Shamim, Upama Mishra, Monika Jain, Khushboo Singh, Jyoti P. Singh, Kavita Dubey, Shweta Singh, Gyanendra K. Rai, Gyanendra P. Singh, Himanshu Pathak, Viswanathan Chinnusamy, Shelly Praveen

**Affiliations:** ^1^Division of Biochemistry, Indian Agricultural Research Institute New Delhi, India; ^2^Department of Molecular Biology and Genetic Engineering, Bihar Agricultural University Bhagalpur, India; ^3^Sher-e-Kashmir University of Agricultural Sciences and Technology Jammu, India; ^4^Indian Institute of Wheat and Barley Research, Indian Council of Agricultural Research Karnal, India; ^5^Central Rice Research Institute Cuttack, India; ^6^Division of Plant Physiology, Indian Agricultural Research Institute New Delhi, India

**Keywords:** terminal HS, wheat, qRT-PCR, protein profiling, starch synthase, total antioxidant capacity, 2-DE, stress-associated proteins

## Abstract

Wheat is highly prone to terminal heat stress (HS) under late-sown conditions. Delayed- sowing is one of the preferred methods to screen the genotypes for thermotolerance under open field conditions. We investigated the effect of terminal HS on the thermotolerance of four popular genotypes of wheat i.e. WR544, HD2967, HD2932, and HD2285 under field condition. We observed significant variations in the biochemical parameters like protein content, antioxidant activity, proline and total reducing sugar content in leaf, stem, and spike under normal (26 ± 2°C) and terminal HS (36 ± 2°C) conditions. Maximum protein, sugars and proline was observed in HD2967, as compared to other cultivars under terminal HS. Wheat cv. HD2967 showed more adaptability to the terminal HS. Differential protein-profiling in leaves, stem and spike of HD2967 under normal (26 ± 2°C) and terminal HS (36 ± 2°C) showed expression of some unique protein spots. MALDI-TOF/MS analysis showed the DEPs as RuBisCO (Rub), RuBisCO activase (Rca), oxygen evolving enhancer protein (OEEP), hypothetical proteins, etc. Expression analysis of genes associated with photosynthesis (*Rub* and *Rca*) and starch biosynthesis pathway (*AGPase, SSS* and *SBE*) showed significant variations in the expression under terminal HS. HD2967 showed better performance, as compared to other cultivars under terminal HS. SSS activity observed in HD2967 showed more stability under terminal HS, as compared with other cultivars. Triggering of different biochemical parameters in response to terminal HS was observed to modulate the plasticity of carbon assimilatory pathway. The identified DEPs will enrich the proteomic resources of wheat and will provide a potential biochemical marker for screening wheat germplasm for thermotolerance. The model hypothesized will help the researchers to work in a more focused way to develop terminal heat tolerant wheat without compromising with the quality and quantity of grains.

## Introduction

Wheat (*Triticum aestivum* L.) is the staple food for the majority of world's populations. Wheat, being highly sensitive to heat stress (HS), is severely affected by the rise in the global temperature due to climate change, and drastic reductions in the yield have been observed in the last few years. HS has been predicted to cause significant reduction in the yield of most of the agriculturally important cereal crops, and predictions on the threat to the future food security are not baseless (Christensen and Christensen, 2007). Reduction of 3–4% in yield has been reported for every 1°C rise in temperature above the ambient, in wheat during the grain-filling stage (Reynolds et al., 1998). HS has, therefore, been predicted as one of the major constraints in wheat production worldwide (Hays et al., 2007; Kumar et al., 2012).

High temperature during the critical reproductive stages—pollination and grain-filling—has drastic effects on the yield of wheat. The genetic built-up of the plant is such that it has two options—escaping the terminal HS, or triggering the expression of stress-associated genes/proteins to protect itself from the elevated temperature. Planting time of wheat can be manipulated to influence the growth, yield and other composition of plants. Wheat is a long-day plant, and longer days in late planting cause shortening of the developmental stages. In late-sown wheat, terminal-HS is the main cause of yield reduction, which is responsible for shortening of grain growth period and improper grain-filling (Rane et al., 2007).

Heat stress (HS) influences the source-to-sink ratio, which, in turn, is reflected in yield reduction. Respiration and photosynthetic rates are altered under the HS, which reduce the plant productivity (Barnabas et al., 2008). Photosynthesis, being highly heat-labile process, is tremendously affected by the HS (Hasanuzzaman et al., 2013). HS also causes scorching of leaves and stems, leaf senescence and growth inhibition etc., which affect the plant yield (Vollenweider and Gunthardt-Goerg, 2005). HS interferes with the shoot net assimilation rate and reduces the kernel weight (Wahid et al., 2007). The reduction in the starch content is the main reason for the yield loss in wheat under the HS (Barnabas et al., 2008). The accumulation of starch is associated with the activities of a series of enzymes like sucrose synthase, sucrose transporter, ADP-glucopyrophosphorylase (AGPase), soluble starch synthase (SSS) etc., which are highly heat-labile (Yan et al., 2008). The low starch content in the endosperm tissue under high temperature stress is mainly because of denaturation / aggregation of starch synthase enzyme which slows down the starch synthesis under the HS (Labuschagne et al., 2009).

The most altered pathway under HS is metabolic pathways followed by secondary metabolite biosynthesis pathway (Kumar et al., 2014). Several gene expression studies suggested that pattern of gene expression under HS might be mediated through signal transduction pathway involving plant hormones, calcium, and sugar molecules, as reported in many plant species (Calderwood et al., 2010). Expression profiling of genes associated with photosynthesis and developing grain is, therefore, of great relevance for elucidating the mechanism of thermotolerance in wheat (Chauhan et al., 2011; Kumar et al., 2013a, 2014).

The present investigation has been undertaken to understand the effect of terminal HS on various biochemical and molecular parameters associated with thermotolerance in contrasting wheat genotypes sown under open field condition. We have given more emphasis to correlate the HS-tolerance with the carbon assimilatory pathway.

## Materials and methods

Seeds of four popular wheat genotypes contrast with respect to thermotolerance—HD2967, HD2932, HD2285, and WR544—were selected from the mini-core subset of wheat developed by Division of Genetics, IARI, New Delhi for the thermotolerance (Table S1). A field experiment was conducted during the year 2012-2013 at the Indian Agricultural Research Institute (IARI), New Delhi. Pre-treated seeds (Bavistin @ 0.25%) were sown at the normal (15th November, 2012) and delayed date (25th December, 2012) under open field condition inside the net-house. The experiment was laid-down in completely randomized block design with split plot arrangement having three replications. The plots were irrigated at uniform intervals and other cultural operations were followed as per the standard farm practices. The samples (leaf, stem and spike) were collected in triplicate at grain-filling stage of growth from both the normal (Max. 26 ± 2°, Min. 12 ± 2°) and delayed sown (Max. 36 ± 2°, Min. 22 ± 2°) plants based on the Feekes scale (Large, 1954) and were immediately frozen in liquid nitrogen for further downstream analysis. The temperature in the net-house was also monitored at regular intervals throughout the experiment (Figure S1). The environmental temperature during sample collection from the normal sown wheat was 26 ± 2° and delayed sown was 36 ± 2° (terminal HS).

### Biochemical screening of selected wheat genotypes for thermotolerance

#### Estimation of total soluble protein (TSP), differential protein profiling and free amino acids

Fresh samples (1 g) of leaves, stem and spike were crushed into fine powder using liq. nitrogen, and transferred into the extraction buffer (Tris-HCl 100 mM, pH 6.8). The homogenate was centrifuged at 32,600 g for 20 min at 4°C and the supernatant was used for the protein estimation by Bradford method (Bradford, 1976); BSA was used for the standard graph preparation. Extracted proteins (15 μg) were separated by SDS-PAGE (Laemmli, 1970) on 12% gel at constant voltage (50 V for 3 h). The gel was stained with 0.1% Coomassie brilliant blue R-250, and destained in 20% methanol and 7.5% (v/v) acetic acid. Total free amino acid was quantified by the method of Moore and Stein (1957) with slight modifications.

#### Estimation of reducing sugars, lipid peroxidation and proline accumulation

Reducing sugars from the fresh leaves, stem, and spike were estimated by the 3, 5-Dinitrosalicylic acid (DNS) colorimetric method (Miller, 1972). Fresh samples (0.1 g) were homogenized in 80% ethanol and centrifuged at 5,000 g for 15 min at room temperature. The supernatant was concentrated in water bath at 80°C. The concentration of reducing sugar was calculated by plotting the unknown OD values on to the graph plotted using glucose as a standard. The lipid peroxidation was calculated as malondialdehyde (MDA) content using Thiobarbituric acid (TBA) following the method of Heath and Packer (1968). Proline was estimated as per the method of Bates et al. (1973).

### Assay of soluble starch synthase (SSS) activity and total antioxidant potential

Soluble starch synthase activity was assayed in endospermic tissue at the grain-filling stage (milky-dough) following the protocol of Nakamura et al. (1989) as modified by Kumar et al. (2013b). Total antioxidant potential was assayed in the fresh leaves as described by Benzie and Strain (1999). The antioxidant potential was expressed as ferric reducing ability of 1 mmol/L FeSO_4_.

### 2D polyacrylamide gel electrophoresis (2D-PAGE) for the identification of differentially expressed stress-associated proteins (SAPs)

The collected samples (leaf, stem and spike) stored in deep freezer (0.5 g) were finely grounded in liquid nitrogen and the total protein was extracted using the Focus Proteome kit (G Bioscience, USA) following the instructions as given by the manufacturer. The protein pellet was re-suspended in 0.4 mL sample buffer [9.5 M urea, 2% (v/v) NP-40, 1% (w/v) DTT and 2% (v/v) 3–10 Biolyte], centrifuged and the supernatant was subjected to two-dimensional electrophoresis (2-DE). The protein concentration was measured by Bradford method. Isoelectric focusing (IEF) was performed using the IEF100 electrophoresis system (Hoefer, USA) and 18 cm SERVA immobilized pH gradient (IPG) blue Strip of 3–10 linear pH gradients (SERVA, Germany). The strips were rehydrated overnight in a solution containing 8 M urea, 2% CHAPS, 20 mM DTT, 0.002% bromophenol blue, 2% IPG buffer (pH 3–10), and 40 μg of the protein sample. IEF was carried out by applying a voltage of 250 V for 1 h, increasing to 3,500 V over 2 h, and holding at 3,500 V until a total of 90 kVh was obtained. Other steps of IEF as well as 1D SDS PAGE were followed as mentioned in Kumar et al. (2013a). The gels were washed in water for 2 h with three changes and placed in 0.02% sodium thiosulfate solution followed by a brief wash and 0.2% silver nitrate solution for 1 h. After brief water wash, the gels were developed in 2% sodium carbonate solution. The gels were stored in 10% acetic acid. The stained gels were scanned using Gel Doc Easy (Bio Rad, USA). Image analysis of the gel was performed using Image Master 2D Platinum (GE Healthcare) version 7.0.6 as per the instructions given by the manufacturer. The spot % volumes (treated/ control) was used for calculating the differentially expressed (fold change) spots between the gels. Ratio more than 1.5 was considered to be upregulated and less than 0.5 as downregulated spots. Spots that did not match in both the gels were considered as unique protein spots.

### MALDI-TOF/TOF MS analysis for the identification of DEPs

The selected spots were manually picked-up one by one using separate 200 μL tip by matching the selected match/spot ID to the actual gel. The selected spots were placed in eppendorf tube containing 10% acetic acid. The silver stained spots were processed for *in gel* digestion, extraction of peptides and drying of samples as mentioned in Kumar et al. (2013b). Samples were analyzed on a MALDI-TOF-TOF mass spectrometer (Ultraflex III, Bruker Daltonics) using an accelerating voltage of 25 kV, 25% laser power and 200 spectra/sec speed for the Peptide Mass Fingerprint (PMF) mode. The Flex analysis 3.3 software (Bruker Daltonics) was used to extract and process the peptide mass peaks from the spectrum. The measured and calibrated tryptic peptide masses were transferred through MS BioTool (Version 3.2, Bruker Daltonics) as inputs to search against the non-redundant NCBI (NCBInr) database using MASCOT 2.2 (Matrix Science) search engine. Search parameters were as follows: Taxonomy: Viridiplantae (green plants); trypsin cleavage; allow up to one missed cleavage; peptide mass tolerance 0.2 Da; fixed modification: carbamidomethyl (C); variable modification: oxidation (M). Protein identifications were accepted if they had greater than 95% probability as represented by the mascot scores in the mascot result page.

### Validation of genes specific to DEPs through quantitative real-time RT-PCR

Total RNA was isolated from the collected samples using the Trizol method (Invitrogen, UK), and quantified by Qubit™ 2.0 Fluorometer (Invitrogen, UK); integrity was verified on 1.2% agarose gel. First strand cDNA synthesis and qRT-PCR were done as mentioned in Kumar et al. (2013a). Primers used for the qRT-PCR were designed from the deduced sequence using GeneFisher2 Prime designing software (http://bibiserv.techfak.uni-bielefeld.de/genefisher2/) (Table 1). The Sybr chemistry (Kappa Sybr Fast Universal kit) was used for the quantification using the CFX96 platform (Bio Rad, UK). Reaction mixture consist of 10 μL of 2x Sybr mix, 0.2 μL of 10 mM primers (forward and reverse), 20 ng of cDNA template and the volume was make up to 20 μL with nuclease free water. The steps followed for the amplification was −98°C for 3 min followed by 40 cycle of 94°C for 20 s, 58°C for 30 s, 72°C for 20 s, and finally plate read. The expression levels of β-actin gene (accession no. AF282624) was used as endogenous control for normalizing the data (Table 1). Relative expression was calculated using Pfaffl method (Pfaffl, 2001).

**Table 1:** List of primers used for the expression analysis of genes associated with photosynthesis (source) and starch biosynthesis pathway (sink) in wheat under normal (26 ± 2°C) and terminal HS (36 ± 2°C) conditions^*^.

**Primers**	**Primer sequence (5′–3′)**	**Tm (°C)**
qRca-F	TACGACATCTCCGATGACCA	60.0
qRca-R	CTCGTAGGAGCTCAGGATGG	59.9
qRbcS-F	GCCGATTGAGGGTATCAAGA	60.0
qRbcS-R	CGAAGCCAACCTTGCTAAAC	59.8
qAGPase-F	CGCAGAGAAACCAAAAGGAG	59.9
qAGPase-R	GAAATTGCTCACGGAGAAGC	59.9
qSSS-F	TGCAAGCTGAAGTTGGATTG	59.9
qSSS-R	CCGGTATGGCTGCAATTAGT	59.9
qSBE-F	AGGAGCAAACGGCTGAAGTA	60.0
qSBE-R	TCCTGGTTTTGGGACAACTC	59.4
β-Act-F	GCGGTCGAACAACTGGTATT	58.4
β-Act-R	GGTCCAAACGAAGGATAGCA	58.4

* *q, Quantitative; f, forward; r, reverse; Rca, RuBisCO activase; RbcS, RuBisCO (small subunit); AGPase, ADP Glucopyrophosphorylase; SSS, soluble starch synthase; SBE, starch branching enzyme; Act, actin, quantitative real-time PCR was used for the expression profiling*.

## Results

### Biochemical screening of wheat genotypes under normal and terminal heat stress

#### Differential protein-profiling

**Figure 1:**
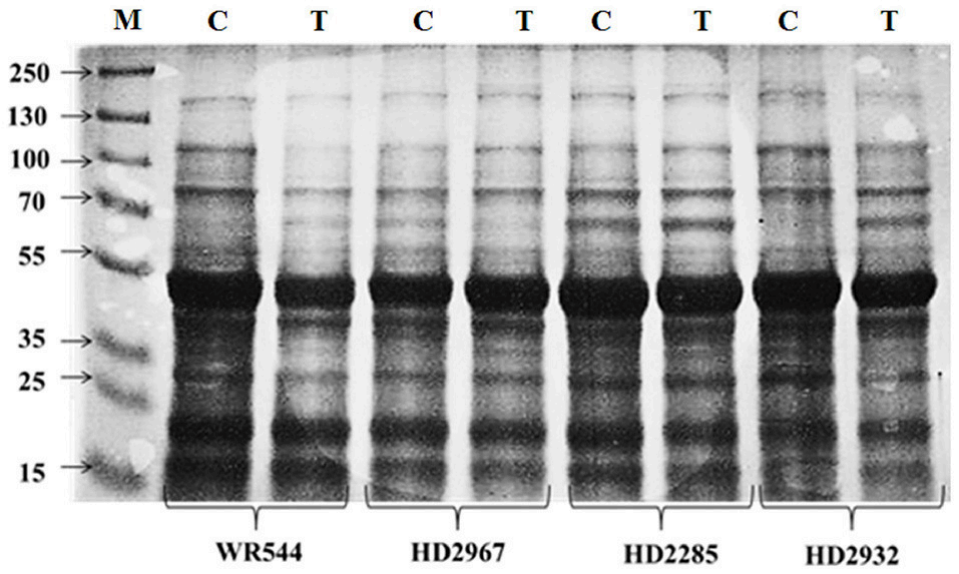
Differential protein profiling of different genotypes of wheat under normal (26 ± 2°C) and delayed sown (36 ± 2°C) conditions. Leaf samples were collected during milky-ripe stage; 12% polyacrylamide gel was used for the SDS-PAGE; NS, normal-sowing; DS, delayed-sowing.

Flag leaf collected from contrasting wheat *cvs*. HD2285, HD2392, HD2967, and WR544 under normal (26 ± 2°C) and terminal HS (36 ± 2°C) conditions were subjected to protein profiling through SDS-PAGE. We observed significant differences in the expression of proteins in all the genotypes; appearance of high molecular weight prominent protein bands were observed in HD2285 and HD2932 and low molecular weight protein bands were observed in HD2967 and WR544 in response to terminal HS (Figure 1). Expression of differentially expressed unique protein bands were observed in case of HD2967 and HD2285 under terminal HS.

### Accumulation of total soluble protein and free amino acid content

**Figure 2:**
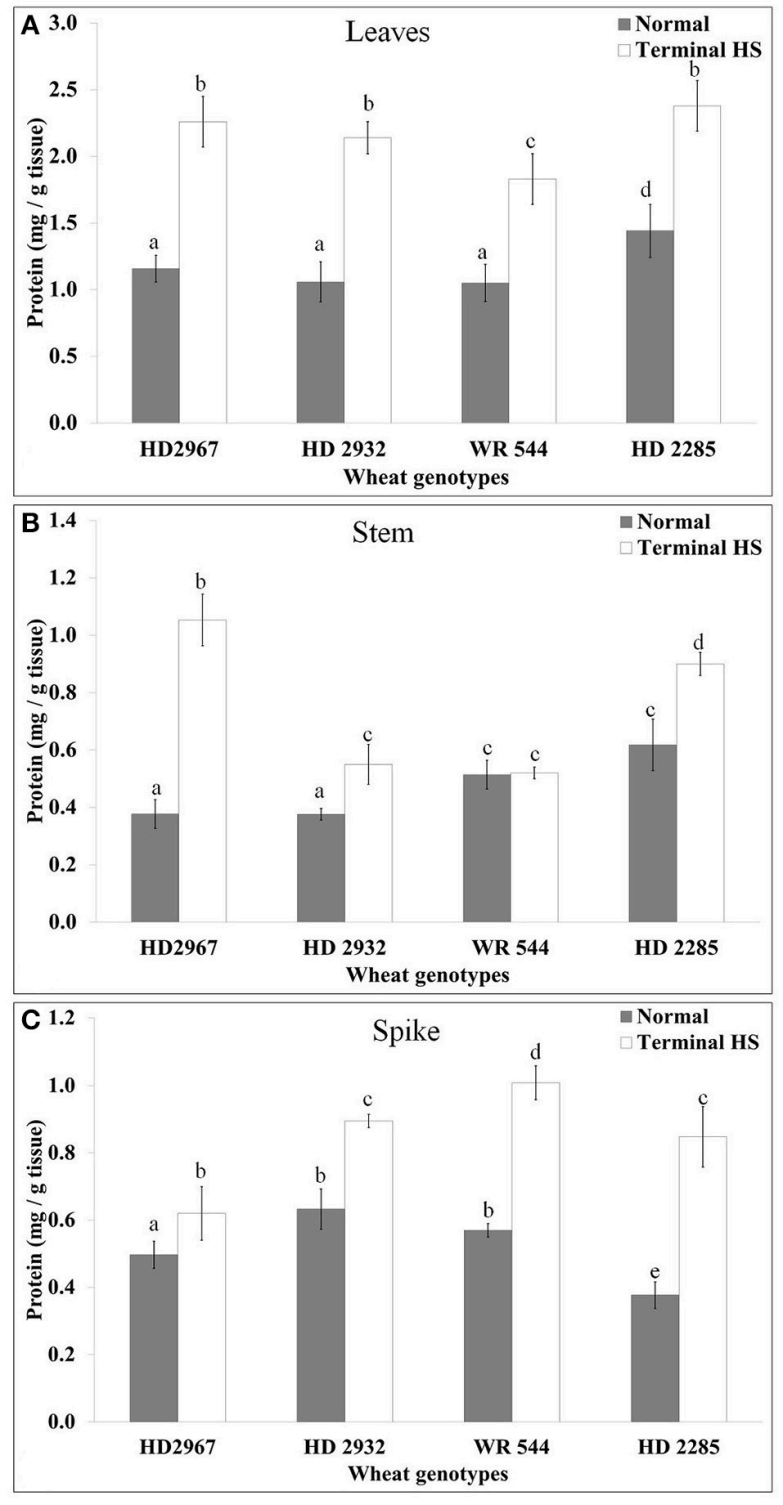
Variations in the protein accumulation in different genotypes of wheat under normal (26 ± 2°C) and delayed-sown (36 ± 2°C) conditions in **(A)** Leaves, **(B)** Stem, and **(C)** Spike. Tissues collected during grain-filling stage were used for the protein estimation by Bradford method; different letters above each bar indicate a significant difference (*p* < 0.05) between treatments (one-way ANOVA); Vertical bars indicate *s.e* (*n* = *3*).

An increase in the total soluble protein was observed in the leaves of different genotypes exposed to terminal HS. The maximum accumulation was observed in HD2967 (thermotolerant) and minimum in WR544 (thermosusceptible) (Figure 2). In stem, maximum accumulation of soluble protein was observed in HD2967 exposed to terminal HS. In spike too, HS caused increased but interestingly highest increase was in the thermosusceptible WR544. It seems different tissues respond differently; percent increase was observed maximum in HD2285 (thermotolerant).

**Figure 3:**
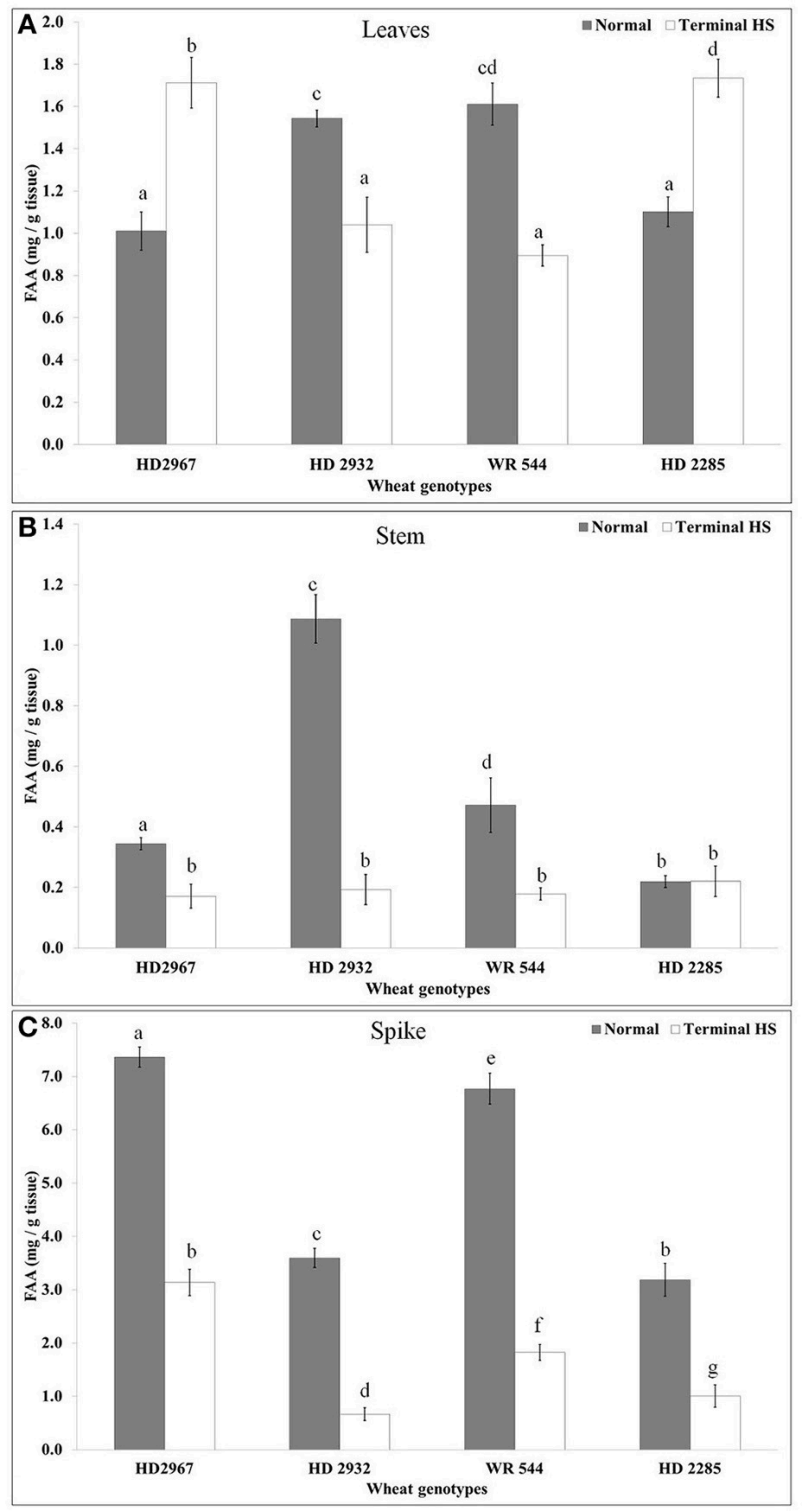
Changes in the Free Amino Acid (FAA) accumulation in different genotypes of wheat under normal (26 ± 2°C) and terminal HS-treated (36 ± 2°C) conditions in **(A)** Leaves, **(B)** Stem, and **(C)** Spike. Thermotolerant—HD2967, HD2285, Thermosusceptible—HD2932, WR544; Leaf, stem and spike collected during grain-filling stage were used for the estimation; Different letters above each bar indicate a significant difference (*p* < 0.05) between treatments (one-way ANOVA); Vertical bars indicate *s.e* (*n* = *3*).

We observed significant increase in the FAA in leaves of thermotolerant *cvs*. (HD2967 and HD2285) under terminal HS, as compared with normal. Maximum FAA was observed in HD2285 (Figure 3). Similarily, thermosusceptible *cvs*. (WR544 and HD2932) showed decrease in the FAA accumulation in leaves under terminal HS, as compared to control; percent decrease was observed maximum WR544.

In case of stem, we observed very less FAA accumulation, as compared to leaves and spikes under normal and terminal HS conditions. Stem of wheat cv. HD2932 showed maximum FAA accumulation under normal condition, whereas drastic decrease was observed under terminal HS condition. Non-significant variations were observed in the FAA of stem of wheat cv. HD2285 under normal and terminal HS-treated conditions. Maximum FAA accumulation was observed in the spike, as compared to leaves and stem under normal and terminal HS-treated conditions. Maximum FAA was observed in the spike of HD2967 followed by WR544 under normal condition, whereas minimum FAA accumulation was observed in HD2932 under terminal HS-treated condition.

### Effect of terminal heat stress on accumulation of reducing sugar

**Figure 4:**
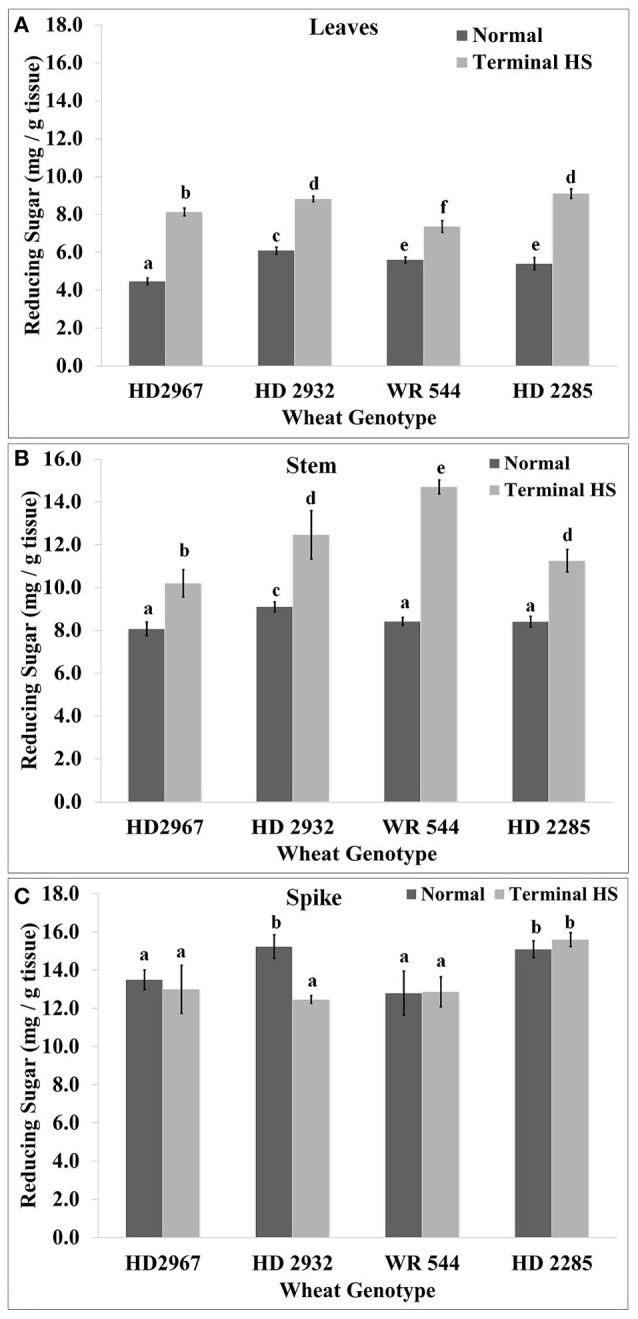
Changes in the reducing sugar accumulation in different genotypes of wheat under normal (26 ± 2°C) and terminal HS-treated (36 ± 2°C) conditions in **(A)** Leaves, **(B)** Stem, and **(C)** Spikes. Tissues collected during grain-filling stage were used for the estimation; HD2967 and HD2285 (thermotolerant) and HD2932 and WR544 (thermosusceptible) were used for the analysis; different letters above each bar indicate a significant difference (*p* < 0.05) between treatments (one-way ANOVA); Vertical bars indicate *s.e* (*n* = *3*).

Reducing sugar content monitored at the mealy-ripe stage showed significantly higher accumulation in the leaves of thermotolerant *cvs*. exposed to terminal HS (36 ± 2°C), as compared with control (26 ± 2°C). Maximum accumulation of reducing sugar in leaves was observed in HD2285 under terminal HS, as compared to control condition. Similarly, minimum sugar accumulation in leaves was observed in WR544 under terminal HS (Figure 4). In stem, maximum reducing sugar was observed in HD2967 exposed to terminal HS, as compared to control. Very low sugar accumulation was observed in the stem of HD2932 under terminal HS, as compared to other *cvs*. In spike, we observed significant decrease in the reducing sugars in terminal HS treated HD2967 and HD2932 *cvs*., as compared with control. HD2285 showed significant increase in the sugar accumulation in spike under terminal HS, as compared to control. WR544 showed non-significant differences in the sugar accumulation in spike under normal and terminal HS conditions (Figure 4).

### Variations in the osmolyte accumulation under terminal HS

**Figure 5:**
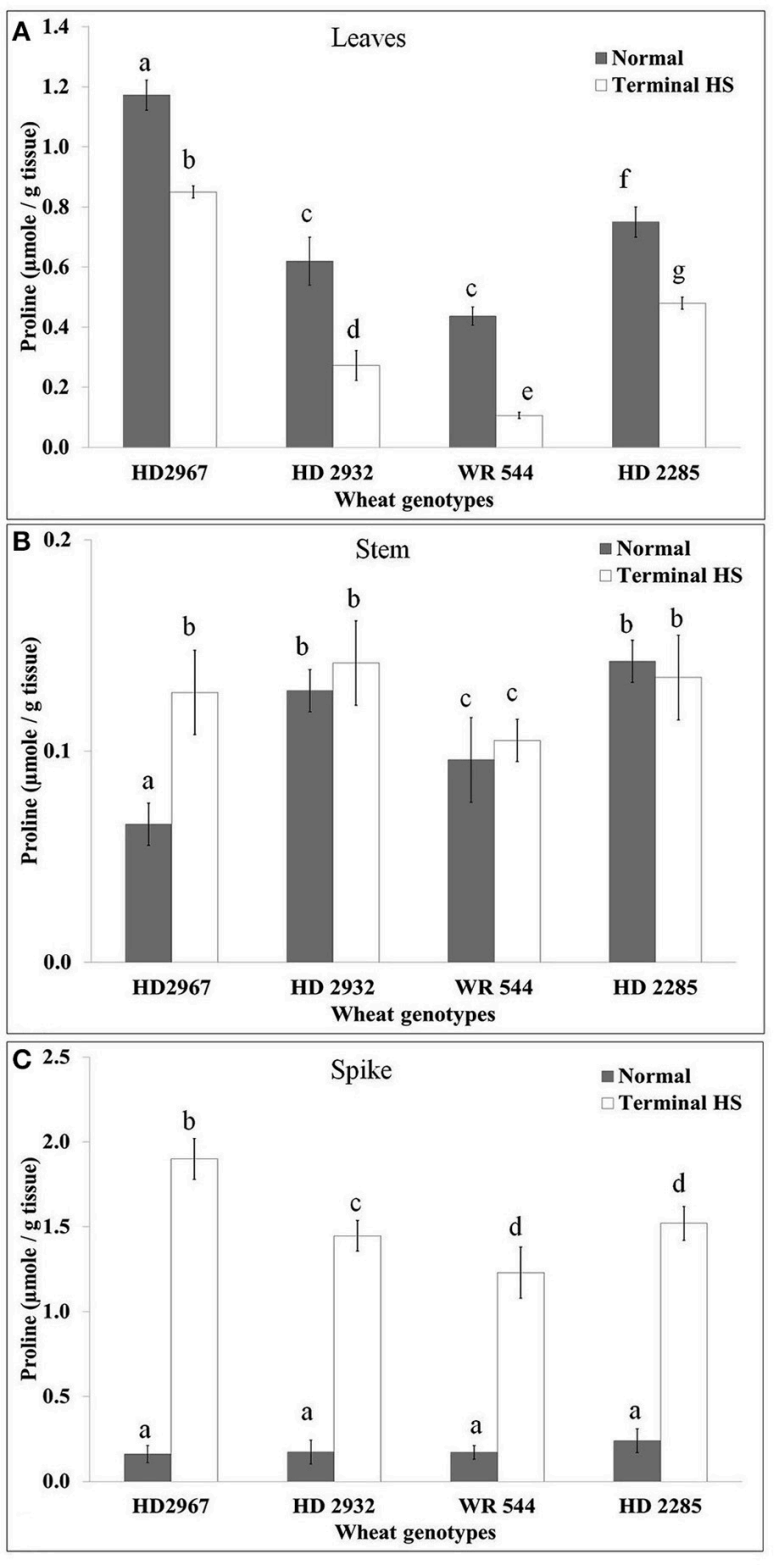
Alteration in lipid peroxidation of different genotypes of wheat under normal (26 ± 2°C) and terminal HS (36 ± 2°C) conditions in **(A)** Leaves, **(B)** Stem, and **(C)** Spikes. Tissues collected during grain-filling stage were used for the estimation; HD2967 and HD2285 (thermotolerant) and HD2932 and WR544 (thermosusceptible) were used for the analysis; Different letters above each bar indicate a significant difference (*p* < 0.05) between treatments (one-way ANOVA); Vertical bars indicate *s.e* (*n* = *3*).

We observed significant reduction in the proline content in the leaves under terminal HS (36 ± 2°C) condition, as compared with normal (26 ± 2°C) in all the cultivars. Percent decrease in the proline accumulation was observed maximum in WR544 (Figure 5). Wheat cv. HD2967 showed maximum accumulation of proline under normal and terminal HS conditions. As against decrease in the proline content in leaves, significant increase was observed in stem of all the *cvs*. under terminal HS. Per cent increase in the proline accumulation under terminal HS was observed maximum in HD2967, as compared to other *cvs*. Spike showed tremendous (>6-fold) increase in the proline in all the *cvs*. under terminal HS; maximum was observed in HD2967 and HD2285 (thermotolerant), respectively.

### Alterations in lipid peroxidation and total antioxidant potential under terminal heat

**Figure 6:**
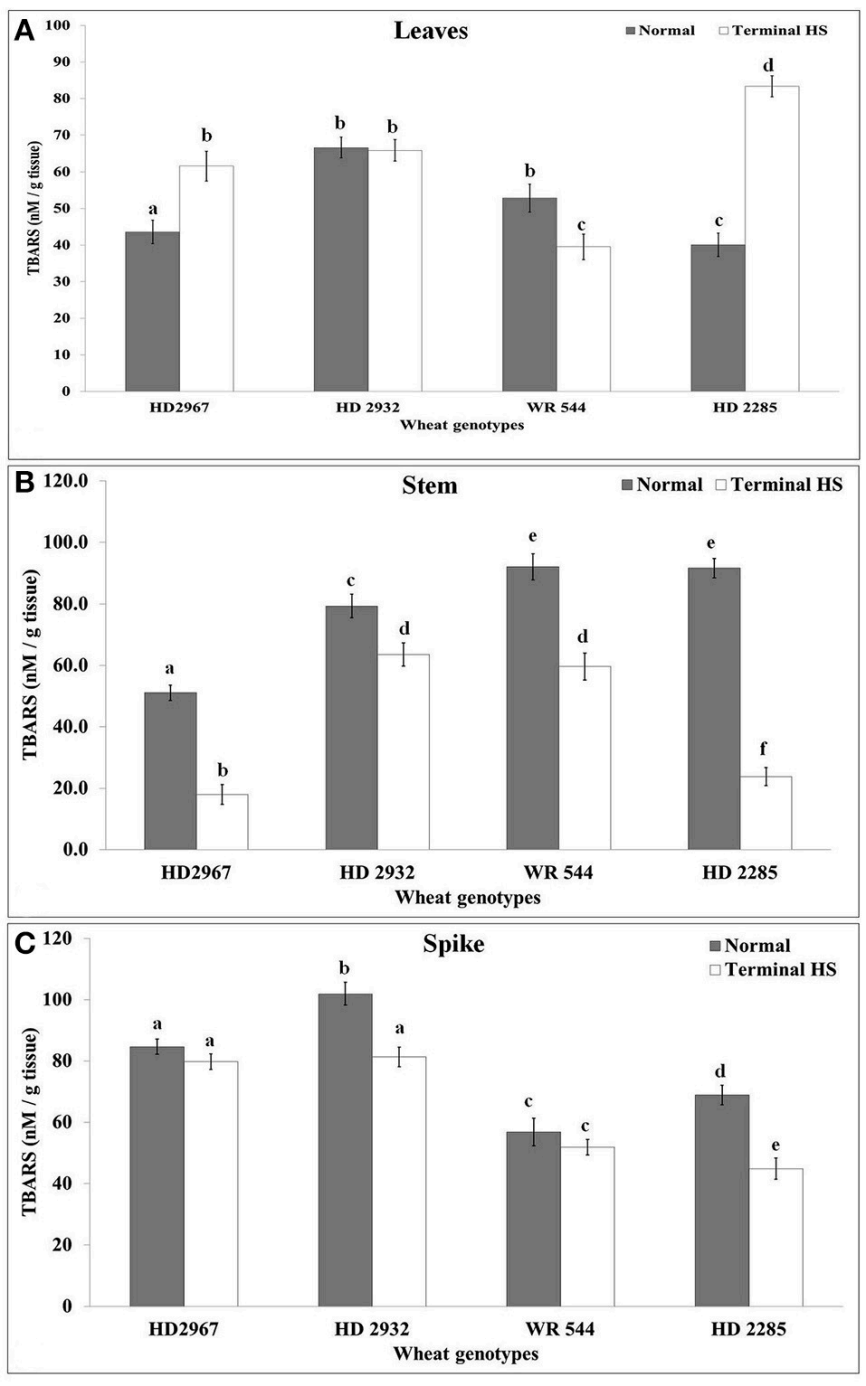
Changes in the Osmolyte accumulation in different genotypes of wheat under normal (26 ± 2°C) and terminal HS (36 ± 2°C) conditions in **(A)** Leaves, **(B)** Stem, and **(C)** Spikes. Thermotolerant—HD2967, HD2285, Thermosusceptible—HD2932, WR544; Leaf, stem and spike collected during grain-filling stage were used for the proline estimation by the method of Bates et al. Different letters above each bar indicate a significant difference (*p* < 0.05) between treatments (one-way ANOVA); Vertical bars indicate *s.e* (*n* = *3*).

Thiobarbituric acid reactive substances were estimated in source (leaf) and sink (stem and spike) of wheat under normal (26 ± 2°C) and terminal HS (36 ± 2°C) conditions. Leaves showed maximum TBARS of 69.5 nM/g tissue in wheat cv. HD2932 under control and 88.5 nM/g tissue in wheat cv. HD2285 under terminal HS condition (Figure 6). Very significant increase in the lipid peroxidation was observed in wheat cv. HD2285 under terminal HS, as compared to normal conditions. Similarly, in case of stem, maximum TBARS (94 nm/g tissue) was observed in wheat cv. WR544 under control and cv. HD2932 (65 nM/g tissue) under terminal HS conditions. We observed significant decrease in the lipid peroxidation in the stem of all the *cvs*. under terminal HS, as compared to normal. We observed similar pattern of decrease in the lipid peroxidation in spike of different wheat *cvs*. under terminal HS; percent decrease was observed maximum in wheat cv. HD2285 under terminal HS, as compared to control.

**Figure 7:**
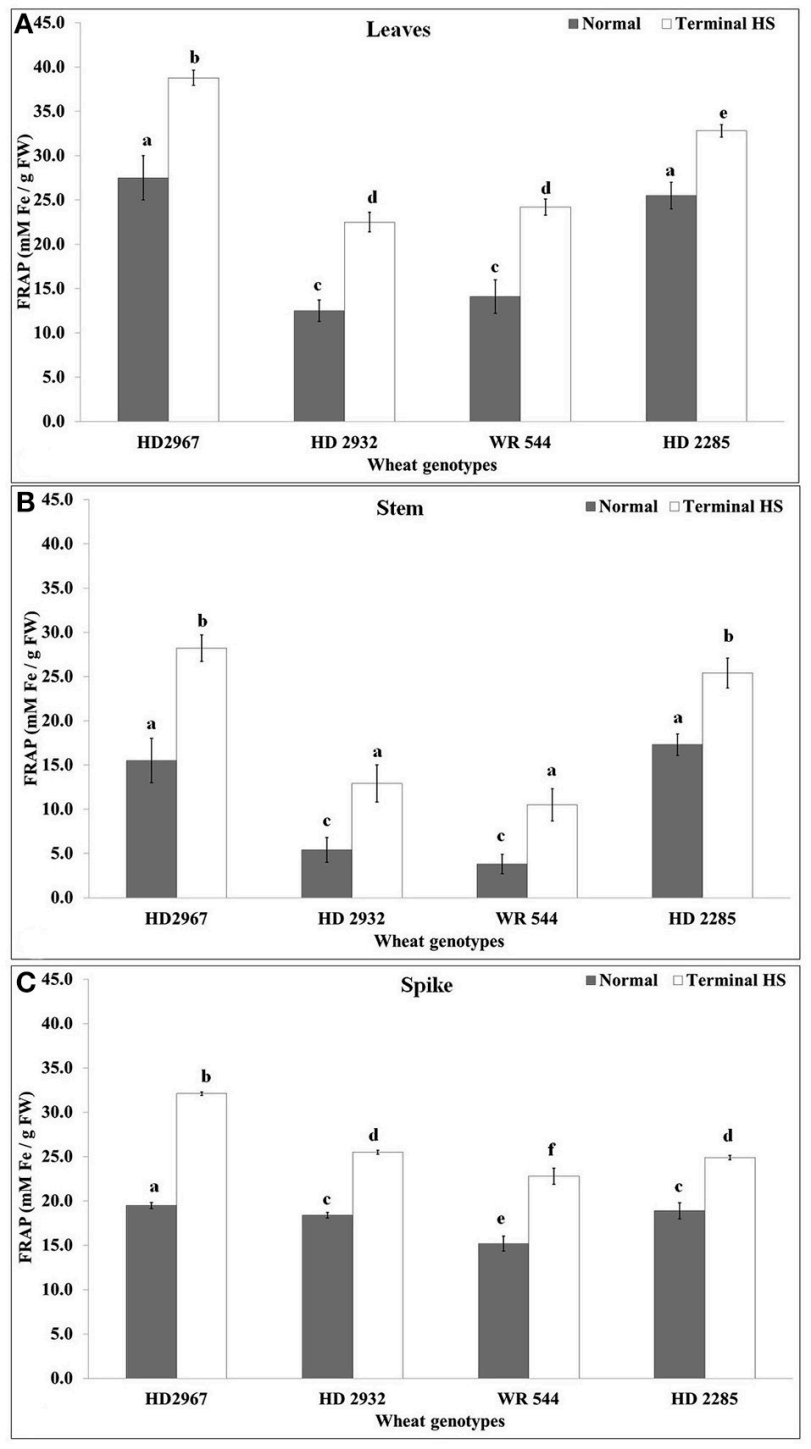
Changes in the total antioxidant capacity (TAC) in different genotypes of wheat under normal (26 ± 2°C) and terminal HS (36 ± 2°C) conditions in **(A)** Leaves, **(B)** Stem, and **(C)** Spikes. Thermotolerant—HD2967, HD2285, Thermosusceptible—HD2932, WR544; Leaf, stem and spike collected during grain-filling stage were used for the estimation; Different letters above each bar indicate a significant difference (*p* < 0.05) between treatments (one-way ANOVA); Vertical bars indicate *s.e* (*n* = *3*).

Total antioxidant capacity represents the tolerance of the crops against abiotic stresses and is used as one of the established biochemical parameters for screening. We observed increase in the antioxidant capacity in leaves of all the *cvs*. under terminal HS (Figure 7). Non-significant differences were observed in the TAC of HD2932 and WR544 under normal and terminal HS conditions. TAC analysis of stem showed maximum ferric reducing potential in wheat cv. HD2285 (18.7 mM Fe2+/g FW) under normal condition, and HD2967 (29.2 mM Fe2+/g FW) under terminal HS-treated condition. Percent increase in the ferric reducing antioxidant potential was observed maximum in the stem of wheat cv. HD2967 in response to terminal HS. FRAP analysis of spike showed maximum (19.2 mM Fe2+/g FW under normal and 33.7 mM Fe2+/g FW under terminal HS) total antioxidant potential in wheat cv. HD2967 (Figure 7). We observed increase in the ferric reducing potential of spike under terminal HS in all the contrasting wheat *cvs*.

### Changes in soluble starch synthase activity under terminal heat

**Figure 8:**
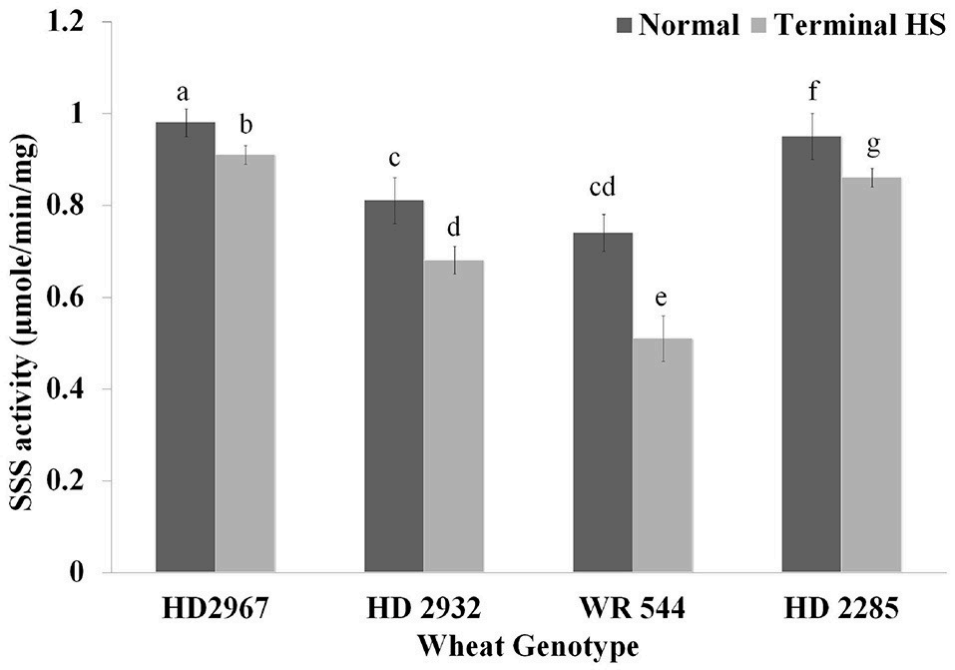
Soluble starch synthase activity assay in different genotypes of wheat under normal (26 ± 2°C) and terminal HS (36 ± 2°C) conditions. Thermotolerant—HD2967, HD2285, Thermosusceptible—HD2932, WR544; Immature endospermic tissue was used for the estimation; Different letters above each bar indicate a significant difference (*p* < 0.05) between treatments (one-way ANOVA); Vertical bars indicate *s.e* (*n* = *3*).

Endospermic tissues collected from wheat *cvs*. under normal and terminal HS conditions were used for the soluble starch synthase (SSS) activity assay. We observed significant decrease in the SSS activity in response to terminal HS; percent reduction in the activity was observed minimum in wheat cv. HD2967. Under normal condition, maximum SSS activity was observed in HD2967 and HD2285, whereas WR544 showed minimum activity. Similar, pattern was observed in samples exposed to terminal HS (Figure 8). SSS present in WR544 was more heat-sensitive, as compared with other cultivars. The activity assay showed the presence of thermostable SSS in HD2967 and HD2285, as compared with other *cvs*.

### Identification and expression of differentially expressed proteins (DEP) spots through 2-DE and MALDI-TOF/TOF MS

**Figure 9:**
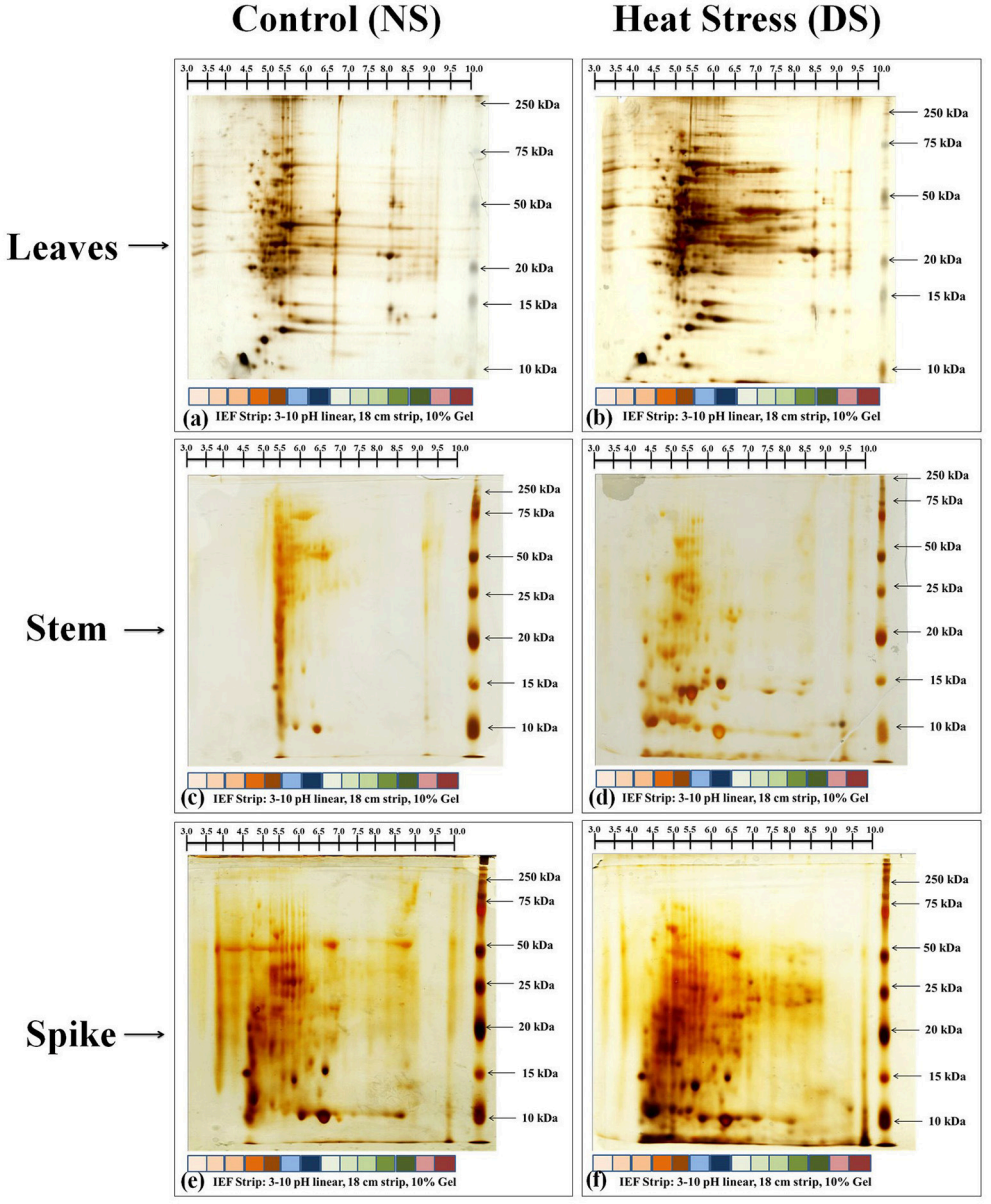
2-DE protein profiling of different tissues of wheat cv. HD2967 under normal (26 ± 2°C) and terminal HS (36 ± 2°C) conditions. **(a)** Gel of leaves under control condition, **(b)** Gel of leaves under HS, **(c)** Gel of stem under control condition, **(d)** Gel of stem under HS, **(e)** Gel of spike under control condition, **(f)** Gel of stem under HS. Leaves, stem and spike were used for the 2-DE; IPG strip of broad range pH from 3.0 to 10 were used for the IEF; RuBisCO clean-up kit was used in case of leaves sample during sample preparation.

**Figure 10:**
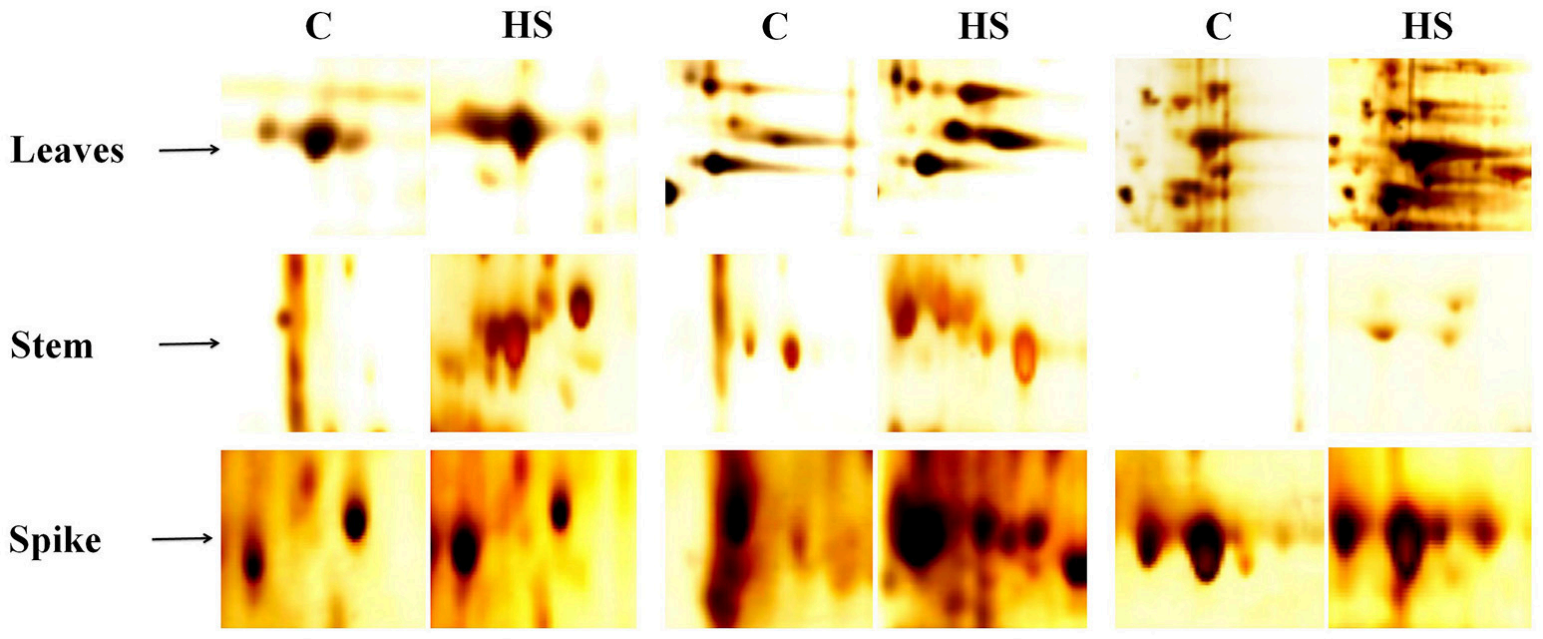
Prominent protein spots targeted for the identification of protein through MALDI-TOF/ TOF MS; Ten protein spots were randomly selected from the gels of leaves, stem and spike under normal (26 ± 2°C) and terminal HS (36 ± 2°C) conditions.

Based on the biochemical screening, we selected thermotolerant genotype (HD2967) for further proteomic characterization and identification of DEPs and their expression analysis. Samples (leaves, stem and spike) collected from HD2967 under normal (26 ± 2°C) and terminal HS (36 ± 2°C) were subjected to 2D PAGE analysis (Figure 9). In case of leaves and spike, we observed expression of many new protein spots, as compared to stem under normal and terminal HS conditions. Leaves of HD2967 showed most of the protein spots lies between the pH of 4.5–8.5. Similarly, in case of stem, we observed maximum protein spots to lie between the pH 5.0–7.0 (normal) and 4.0–8.5 (terminal HS). Gels of spike showed maximum diversity with protein spots spread up between pH 4.0–9.0 (Figure 9). Some of the prominent protein spots were picked through IMP7 software from the leaves, stem and spike (Figure 10).

2-DE gel analysis of leaves under control (26 ± 2°C) and terminal HS (36 ± 2°C) showed 2 upregulated (ratio >1.5) and 14 down-regulated (ration <0.5) DEP spots (Table 2). We observed maximum downregulation of 0.3- fold (spot 3) and up-regulation of 1.49-fold (spot 9) in leaves of wheat cv. HD2967 under terminal HS. Similarly, 2-DE analysis of stem showed 8 upregulated (ration > 1.5) and 1 downregulated (ration < 0.5) DEP spots under terminal HS. Maximum upregulation was observed in spot 9 (6.6-fold), whereas downregulation was observed in case of spot 3 (0.9-fold). In case of spikes, we observed 24 DEP spots with 11 upregulated (ration > 1.5) and 13 downregulated (ration < 1.5) under terminal HS. Maximum upregulation was observed in protein spot 40 (8.65-fold) and downregulation in case of spot 35 (0.13-fold) under terminal HS (Table 2).

**Table 2:** Expression analysis of differentially expressed protein (DEP) spots in different tissues of wheat cv. HD2967 under terminal heat stress (36 ± 2°C).

**S. No**.	**Match ID**	**Fold increase**
**LEAVES**		
**Over-expressed spots**		
1	0	1.59
2	7	1.92
**Under-expressed spots**		
3	3	0.30
4	15	0.31
5	10	0.33
6	1	0.43
7	4	0.49
8	12	0.50
9	13	0.51
10	11	0.57
11	8	0.63
12	6	0.80
13	14	0.85
14	5	1.07
15	2	1.18
16	9	1.49
**STEM**		
**Over-expressed spots**		
1	6	1.53
2	8	1.69
3	4	2.02
4	5	2.04
5	1	2.41
6	2	2.87
7	7	4.65
8	9	6.66
**Under-expressed spots**		
9	3	0.97
**SPIKES**		
**Over-expressed spots**		
1	17	1.61
2	4	1.75
3	41	1.92
4	37	2.10
5	10	2.14
6	23	2.15
7	48	3.89
8	20	5.47
9	1	5.70
10	33	6.39
11	40	8.65
**Under-expressed spots**		
12	35	0.13
13	39	0.18
14	51	0.25
15	22	0.26
16	30	0.34
17	11	0.35
18	2	0.42
19	47	0.46
20	27	0.47
21	7	0.48
22	16	0.50
23	24	0.50
24	38	0.51

We randomly selected 10 DEP spots from leaves, stem and spikes of wheat cv. HD2967 for establishing their identity through MALDI-TOF/ TOF MS (Table 3). Based on the m/Z ratio and mascot search analysis, the DEPs were identified as RuBisCO (small subunit; spot ID 25), oxygen evolving enhancer protein (spot ID 14), ATP synthase alpha subunit (spot ID 19), chloroplast fructose-bisphosphate aldolase (spot ID 12), dehydration-responsive protein (spot ID 36) and other predicted and hypothetical proteins (Table 3).

**Table 3:** Identification of randomly selected differentially expressed proteins in different tissues of wheat cv. HD2967 under terminal heat stress (36 ± 2°C) condition.

**Protein spot ID**	**Accession no**.	**Protein name**	**Peptide sequence**
13	gi|326488091	predicted protein [*Hordeum vulgare* subsp. vulgare]	R.QVQCVSFIAFRPPGCEESGKA.
25	gi|11990895	ribulose-1,5-bisphosphate carboxylase/oxygenase small subunit [*Triticum aestivum*]	K.LPMFGCTDATQVINEVEEVK.K
14	gi|357111487	PREDICTED: oxygen-evolving enhancer protein 1, chloroplastic-like isoform 2 [*Brachypodium distachyon*]	K.QLVATGKPESFSGPFLVPSYR.G
36	gi|290760321	dehydration-responsive protein RD22 precursor [*Linum usitatissimum*]	K.VMNLDFTETPQR.A
22	gi|739292	oxygen-evolving complex protein 1	K.DGIDYAAVTVQLPGGER.V
19	gi|194033146	ATP synthase CF1 alpha subunit [*Brachypodium distachyon*]	R.LIESPAPSIISRR.S
42	gi|552976	ATP synthase alpha subunit [*Triticum aestivum*]	K.GEIIASESRLIESPAPSIISR.R
8	gi|125531809	hypothetical protein OsI_33462 [*Oryza sativa* Indica Group]	R.QEAANSYHYYYCYKPSLAASYR.A
12	gi|223018643	chloroplast fructose-bisphosphate aldolase [*Triticum aestivum*]	K.TWGGRPENVAAAQEALLLR.A
27	gi|357161336	PREDICTED: ATP-citrate synthase alpha chain protein 3-like [*Brachypodium distachyon*]	R.LGCTISFSECGGIDIEENWDK.V

### Expression of photosynthetic pathway-associated genes under terminal HS

**Figure 11:**
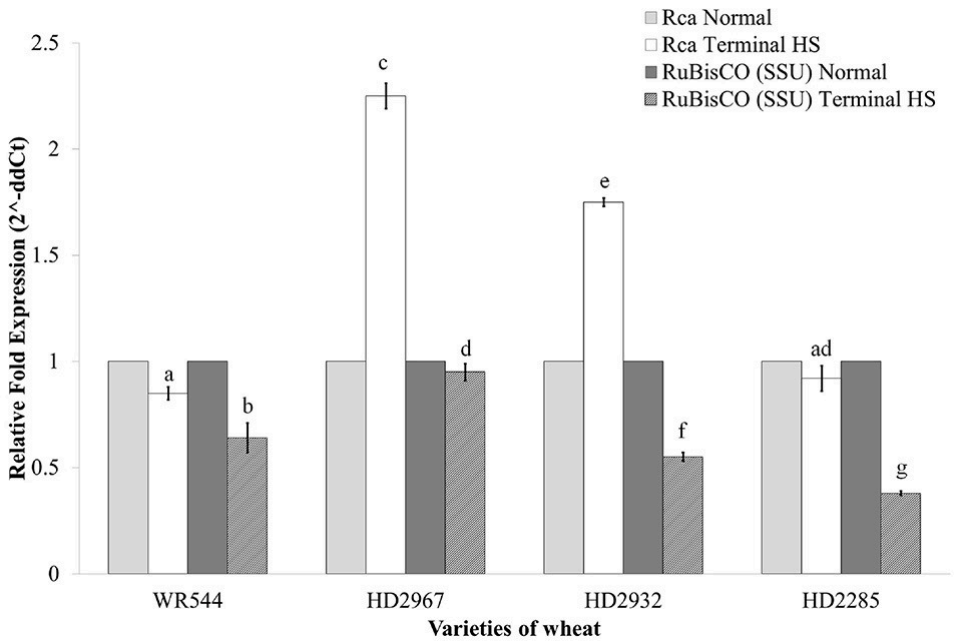
Expression profiling of genes associated with photosynthesis in different genotypes of wheat under normal (26 ± 2°C) and terminal HS-treated (36 ± 2°C) conditions. RuBisCO and RuBisCO activase genes were used for the expression profiling; β- actin gene was used as endogenous control for normalizing the data; different letters above each bar indicate a significant difference (*p* < 0.05) between treatments (one-way ANOVA); Vertical bars indicate *s.e* (*n* = *3*).

We analyze the expression of two key genes associated with photosynthesis *i.e*. RuBisCO (small subunit, *rbcS*; acc. no. AB020941.1) and RuBisCO activase (*Rca*; acc. no. KC776912) under terminal HS (36 ± 2°C) in contrasting wheat *cvs*. during milky-ripe stage (Figure 11). *Rca* showed maximum relative fold expression (2.38-fold) in terminal HS-treated HD2967, as compared to normal condition (Figure 11). Similarly, the relative fold expression of *Rca* was observed minimum in WR544 (0.75-fold) in response to terminal HS, as compared to normal condition. We observed upregulation of *Rca* in thermotolerant cultivars, and downregulation in thermosusceptible cultivars under terminal HS.

Expression analysis of *rbcS* showed downregulation in all the four cultivars in response to terminal HS, as compared to normal condition. Percent decrease was observed maximum in HD2285 and minimum in HD2967 under terminal HS (Figure 11).

### Expression of starch biosynthesis pathway-associated genes under terminal HS

**Figure 12:**
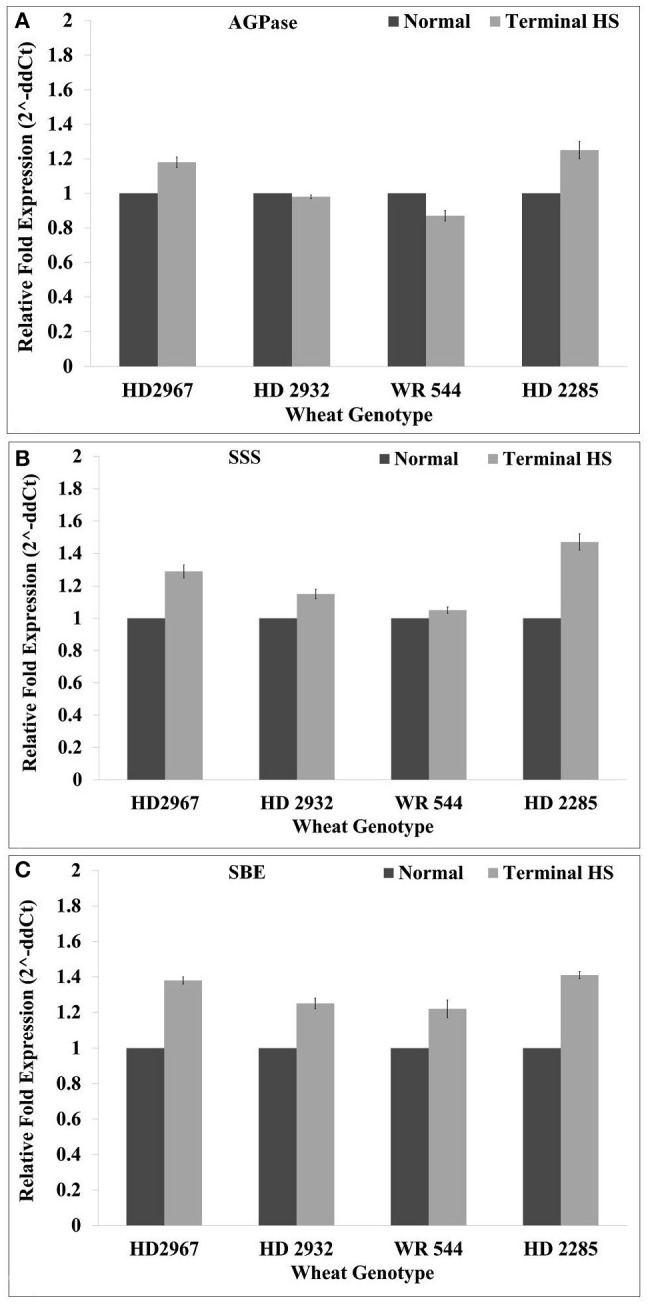
Expression profiling of starch biosynthesis pathway-associated genes in different genotypes of wheat under normal (26 ± 2°C) and terminal HS-treated (36 ± 2°C) conditions. **(A)** Relative fold expression of AGPase, **(B)** relative fold expression of SSS, and **(C)** relative fold expression of SBE. ADP glucophosphorylase (*AGPase*) (acc. no. X66080.1), soluble starch synthase (*SSS*) (acc. no. KM206143) and starch branching enzyme (*SBE*) (acc. no. AF338432.1) were used for the expression profiling; β-actin gene was used as endogenous control for normalizing the data; Vertical bars indicate *s.e* (*n* = *3*).

The expression of key genes associated with starch biosynthesis pathway—ADP glucopyrophosphorylase (*AGPase*; acc. no. X66080.1), soluble starch synthase (*SSS*; acc. no. KM206143) and starch branching enzyme (*SBE*; acc. no. AF338432.1) genes—were analyzed for their expression in wheat under terminal HS (36 ± 2°C), as compared with control during milky-ripe stage (Figure 12). The relative expression of *AGPase* was observed very high (1.28-fold) in terminal HS-treated wheat cv. HD2285, as compared to normal condition. We observed downregulation of *AGPase* in HD2932 and WR544 in response to terminal HS (Figure 12). Similarly, *SSS* showed maximum relative expression (1.5-fold) in terminal HS-treated wheat cv. HD2285, as compared to normal condition. The expression of *SSS* in WR544 was observed very low in response to terminal HS. Similar pattern of expression of *SBE* was observed in contrasting wheat *cvs*; maximum relative fold expression was observed in HD2285 (1.42-fold) under terminal HS.

### Model hypothesized for elucidating the plasticity of source and sink under normal and terminal HS

**Figure 13:**
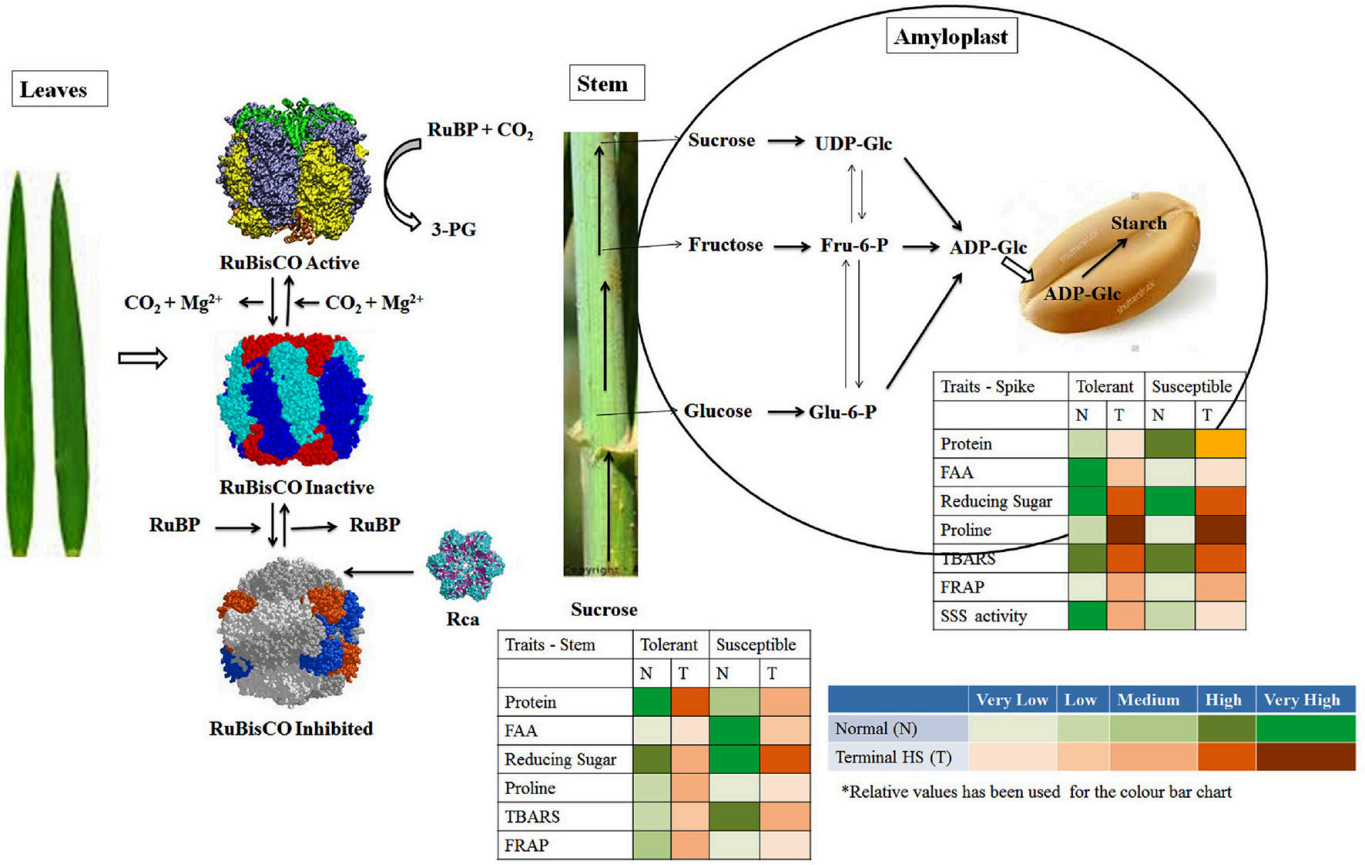
Model hypothesized for characterizing the plasticity of carbon assimilatory pathways in contrasting wheat cultivars under normal (26 ± 2°C) and terminal HS (36 ± 2°C) conditions. Different tissues (leaves, stem and spike) collected from contrasting wheat *cvs*. under normal and terminal HS-treated conditions were used for the biochemical analysis.

Photosynthesis is the key biochemical process involved in carbon fixation in plants and is highly sensitive to HS. Even slight fluctuation in temperature causes drastic reduction in the photosynthetic rate which alters the source to sink ratio and ultimately shriveled seeds are formed. A network of different biochemical parameters are involved in modulating the tolerance level of plant under stresses. In present investigation, we observed very high accumulation of proteins in leaves of tolerant cultivars under normal (26 ± 2°C) and terminal HS (36 ± 2°C), whereas low accumulation was observed in case of thermosusceptible cultivars. Similarily, TBARS, FRAP and Rca activity was observed very high in the leaves of thermotolerant cultivars exposed to terminal HS, as compared to normal condition (Figure 13). In case of thermosusceptible cultivars, except FAA (under normal condition) and FRAP (under terminal HS), all other biochemical parameters showed very low accumulation in leaves under normal and terminal HS conditions.

Stem harbors different transporters and enzymes associated with the movement of photosynthates from leaves to the endospermic tissue. Sucrose is transported via phloem and part of it is accumulated in the stem as reserve. Sucrose also gets hydrolyzed to glucose and fructose, which is further utilized for the synthesis of glucan chain. We observed very high accumulation of total soluble protein in stem under normal and terminal HS conditions (Figure 13). All other biochemical parameters, except reducing sugar showed low accumulation in stem under above mentioned conditions. FRAP which signifies the tolerance level of the tissue was observed medium under normal and terminal HS conditions.

Cytosol followed by amyloplast has all the enzymes involved in starch biosynthesis in endospermic tissue. These enzymes involved in starch biosynthesis are highly heat labile. ADP-Glc enters inside the amyloplast, where it is acted upon by AGPase followed by SSS and SBE for the synthesis of starch. In case of thermotolerant cultivars, we observed very high accumulation of FAA, reducing sugar and high activity of SSS in amyloplast under normal conditions (Figure 13). Similarly, reducing sugar, Proline and TBARS was observed very high in amyloplast under terminal HS, as compared to normal condition. All other parameters like protein, FRAP, etc. were observed very low to medium under normal and terminal HS conditions. In case of thermosusceptible cultivars, we observed very high accumulation of protein, reducing sugar and TBARS in amyloplast under normal and terminal HS conditions. Very low FAA, FRAP and SSS activity was observed in the amyloplast under terminal HS.

## Discussion

Wheat is highly heat-sensitive and even slight fluctuation in environmental temperature during critical stages severely affects the growth, development and yield of the crop (Kurek et al., 2007). Wheat has not much been explored because of the complexity of the genome and availability of limited information about genes/ proteins on public domain (Kumar et al., 2014). In spite of so much importance, most of the key pathways associated with source (photosynthesis) and sink (starch biosynthesis) has not been unraveled. Researchers are still struggling hard to find out the missing links (genes/ enzymes) in the above mentioned pathways, so that they can be utilized to develop a “climate-smart” wheat crop.

Here, we characterized the contrasting genotypes (HD2967, HD2932, WR544, and HD2285) of wheat for thermotolerance by exposing them to normal and terminal HS conditions. Protein profiling through 1D-SDS-PAGE showed expression of many new protein bands in terminal HS-treated samples, as compared to normal. These DEPs are predicted to be SAPs as reported earlier in wheat from our lab (Kumar et al., 2014). The selected genotypes were screened using different biochemical parameters like protein accumulation, total soluble sugar, TAP, lipid peroxidation, osmolyte accumulation, etc. We observed significant increase in the total soluble protein under terminal HS (36 ± 2°C) condition, as compared with normal (26 ± 2°C). This may be due to the over-expression of SAPs under terminal HS, which triggers the defense mechanism of the plant. Leaves showed maximum accumulation of total proteins in all the four cultivars under terminal HS, compared with stem and spike. The findings are in conformity with the observation of Kumar et al. (2013b) and Leonardis et al. (2015). Wheat *cvs*. HD2967 and HD2285, being thermotolerant, showed maximum accumulation of total soluble protein compared to other *cvs*. The accumulation of total soluble protein in the different tissues (leaves, stem and spike) under terminal HS might be playing role in the defense response of the plant. Accumulation of total protein was observed more in the leaves and stem of thermotolerant cultivars (HD2967 and HD2285), as compared to thermosensitive (WR544 and HD2932) under normal and terminal HS conditions. Spike of WR544 showed maximum accumulation of total soluble protein under terminal HS condition. This may be the reason behind the high protein content in the grains of WR544, as compared to other *cvs*. Our findings are in conformity with the observation of Asthir and Bhatia (2014) who reported increase in the accumulation of total free sugars and protein content in the grains of contrasting wheat cultivars under HS. They also reported that the grain might responds to the HS mediated alteration in carbon assimilatory pathway by a compensatory effect on nitrogen metabolism.

A significant increase in the FAA was observed in the leaves of thermotolerant *cvs*. exposed to terminal HS, as compared with thermosensitive. The disruption in the protein synthesis or partial hydrolysis may be the reason behind this. In other tissues like stem and spike, we observed decrease in the FAA under terminal HS in both the thermotolerant as well as thermosensitive *cvs*. The findings are in conformity with the observation of Leonardis et al. (2015) in wheat. Lipid peroxidation is used as one of the biochemical traits for screening crop for abiotic stress tolerance. A significant increase in the TBARS content was observed in wheat *cvs*. exposed to terminal HS (36 ± 2°C), as compared to control (26 ± 2°C). High temperature has severe effect on the membranic lipids, as it gets oxidized with the extent of the stress causing damage of membrane and the cells as such. Our findings are in concordance with the observation of Almeselmani et al. (2006) and Goswami et al. (2016).

We observed increase in the accumulation of proline in wheat *cvs*. exposed to terminal HS, compared to control. The high concentration of proline might be playing role in osmotic balance. Wang et al. (2014) reported that proline accumulation regulates protein synthesis and the cell cycle transition in plants. Increase in the proline under stresses has been reported in different crops (Jain et al., 2013). Total antioxidant potential which signifies the tolerance level of the plant under stress was observed very high in thermotolerant cultivars, as compared to susceptible. We observed increase in the TAP of the plant under terminal HS and the percent increase was observed more in tolerant *cvs*., compared with thermosusceptible. Leonardis et al. (2015) observed increase in the TAP in seed grain of Durum under HS. Similarly, Sairam and Srivastava (2002) reported increase in the total antioxidant activity in tolerant and susceptible wheat cultivars in wheat exposed to abiotic stress.

Cultivars exposed to terminal-HS during grain-filling showed reduction in the activity of starch biosynthesis pathway-associated enzymes. We observed decrease in the activity of SSS under terminal HS, which is in conformity with our earlier observation (Kumar et al., 2013b). SSS is highly heat-labile (Kumar et al., 2014). DuPont et al. (2006) reported that HS during grain-filling decreases the duration of grain-filling and reduces the dry matter accumulation and kernel weight. It has been reported that stem reserves before grain-filling is considered as an important source of carbon required for grain filling, especially when plant is exposed to heat, drought or any biotic stresses. Prolonged HS has been reported to starve the plants by utilizing the stem reserve (Hasanuzzaman et al., 2013).

In order to identify the DEPs, we used the 2-DE followed by MALDI-TOF-MS in different tissues of wheat cv. HD2967. We observed the expression of unique and DEPs in leaf, stem and spike of HD2967 under terminal HS (36 ± 2°C), which is in conformity with our earlier observation (Kumar et al., 2013b). High and low molecular weight proteins were observed abundant under terminal HS-treated wheat, which may be predicted to be HSPs, signaling molecules, etc. as reported earlier by different researchers in wheat. Wang et al. (2015) have reported that proteins related to heat shock, photosynthesis, stress defense, glycolysis, signaling, etc. are differently expressed in leaves which causes lower damage to the cell membranes in tolerant compared to sensitive cultivars under the HS.

MALDI-TOF/MS of 10 randomly selected DEP spots showed the presence of RuBisCO, ATP Synthase, Oxygen evolving enhancer protein, hypothetical proteins, etc. The findings are in conformity with our earlier observation in wheat exposed to HS of 42°C for 2 h (Kumar et al., 2014). The identified DEPs were mostly involved in photosynthesis, transport of photosynthates and starch biosynthesis pathway. The identified hypothetical proteins are predicted to act as enzymes in pathways associated with source and sink.

Terminal heat causes upregulation of ~5% of the transcript in plants (Saidi et al., 2011). Under HS, transcriptional regulation has been predicted to inhibit the starch metabolism (Cossani and Reynolds, 2012). We observed downregulation in the expression of *AGPase* in response to terminal HS (36 ± 2°C), as compared with normal condition (26 ± 2°C). *SSS* and *SBE* showed upregulation in all the *cvs*. under terminal HS. AGPase and SSS have been established as the regulatory enzymes of starch biosynthesis pathway (Glaring et al., 2012).

To conclude, Terminal HS modulates the various biochemical and molecular parameters associated with thermotolerance in wheat. The effect was observed more pronounced in HD2285 and HD2967 (thermotolerant). These varieties can be used as good genetic stocks for the breeding program in order to develop high yielding wheat cultivars under elevated temperature.

## Author contributions

RK, SG, and SP conceptualized the ideas and lay down the experiment; JS and KD collected the samples; MS, UM, and MJ performed the biochemical characterization of the collected samples; JS, KS, and KD performed the real-time PCR for the expression analysis; SS and GR executed the activity assay of SSS; RK, GS, and VC drafted the manuscript; GS, VC, HP, and SP edited the manuscript.

### Conflict of interest statement

The authors declare that the research was conducted in the absence of any commercial or financial relationships that could be construed as a potential conflict of interest.

## Supplementary material

Figure S1 Mean daily temperature (maximum and minimum) profiling of experimental site during the period of the crop growth.

Table S1 Summary of characteristic of spring wheat cultivars selected for analyzing the effect of delayed-sowing on thermotolerance. Based on field trial, HD2285 and HD2967 have been predicted as thermotolerant and HD2932 and WR544 as thermosusceptible.

## Abbreviations

qRT-PCR: Quantitative real-time PCR
THS: terminal heat stress
SSS: soluble starch synthase
SAP: stress-associated proteins
DEPs: differentially expressed proteins
2-DE: two dimensional electrophoresis.
