# Supplementary figures and images for: Biochemical Defense Response: Characterizing the Plasticity of Source and Sink in Spring Wheat under Terminal Heat Stress
Source: Front Plant Sci. 2017 Sep 20;8:1603. doi: 10.3389/fpls.2017.01603 (PMC5611565; doi:10.3389/fpls.2017.01603)

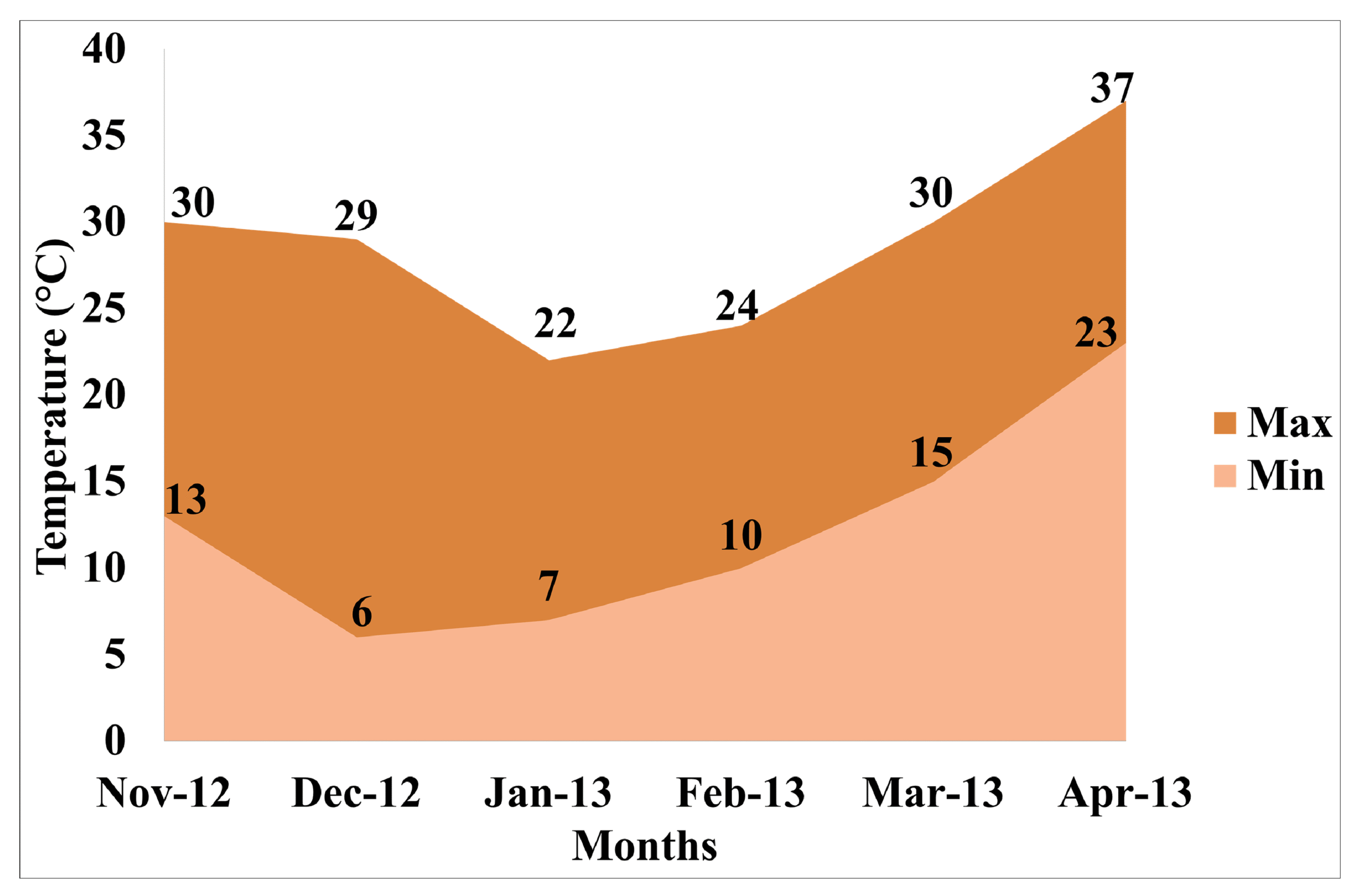

Supplement: Figure S1 — Mean daily temperature (maximum and minimum) profiling of experimental site during the period of the crop growth. [file Image1.TIF]
