# Supplementary material for: Biochemical Defense Response: Characterizing the Plasticity of Source and Sink in Spring Wheat under Terminal Heat Stress
Source: Front Plant Sci. 2017 Sep 20;8:1603. doi: 10.3389/fpls.2017.01603 (PMC5611565; doi:10.3389/fpls.2017.01603)
Supplement: Table S1 — Summary of characteristic of spring wheat cultivars selected for analyzing the effect of delayed-sowing on thermotolerance. Based on field trial, HD2285 and HD2967 have been predicted as thermotolerant and HD2932 and WR544 as thermosusceptible. [file Table1.DOCX]

**Table S1.** Summary of characteristic of spring wheat cultivars selected for analyzing the effect of delayed-sowing on thermotolerance. Based on field trial, HD2285 and HD2967 has been predicted as thermotolerant and HD2932 and WR544 as thermosusceptible

| **Variety** | **Parentage** | **Special characters** |
| --- | --- | --- |
| WR544 (Pusa Gold) | Kalyansona/HD1999//HD 2204 /DW 38 | Late sowing variety |
| HD2967 | ALD/COC//URES/HD2160M/HD2278 |  |
| HD2285 (Gobind) | 249/HD2150 //HD 2186 | Suitable for very early sown, late sown and irrigated conditions |
| HD2932 (Pusa wheat 111) | Kauz/Star//HD 2643 |  |
